# Parental Smoking Modifies the Relation between Genetic Variation in Tumor Necrosis Factor-α (*TNF*) and Childhood Asthma

**DOI:** 10.1289/ehp.9740

**Published:** 2007-01-16

**Authors:** Hao Wu, Isabelle Romieu, Juan-Jose Sienra-Monge, Blanca Estela del Rio-Navarro, Daniel M. Anderson, Erin W. Dunn, Lori L. Steiner, Irma del Carmen Lara-Sanchez, Stephanie J. London

**Affiliations:** 1 Laboratory of Respiratory Biology, Division of Intramural Research, National Institute of Environmental Health Sciences, National Institutes of Health, Department of Health and Human Services, Research Triangle Park, North Carolina, USA; 2 National Institute of Public Health, Cuernavaca, Morelos, Mexico; 3 Hospital Infantil de Mexico Federico Gomez, Mexico City, Mexico; 4 Department of Human Genetics, Roche Molecular Systems, Alameda, California, USA; 5 Epidemiology Branch, Division of Intramural Research, National Institute of Environmental Health Sciences, National Institutes of Health, Department of Health and Human Services, Research Triangle Park, North Carolina, USA

**Keywords:** allergy, asthma, atopy, environmental tobacco smoke, genetic predisposition to disease, lymphotoxin-α (LTA), ozone, secondhand smoke, single nucleotide polymorphism (SNP), tumor necrosis factor-α (TNF)

## Abstract

**Background:**

Polymorphisms in the proinflammatory cytokine genes tumor necrosis factor-α (*TNF*) and lymphotoxin-α (*LTA*, also called *TNF-*β) have been associated with asthma and atopy in some studies. Parental smoking is a consistent risk factor for childhood asthma. Secondhand smoke and ozone both stimulate TNF production.

**Objectives:**

Our goal was to investigate whether genetic variation in *TNF* and *LTA* is associated with asthma and atopy and whether the association is modified by parental smoking in a Mexican population with high ozone exposure.

**Methods:**

We genotyped six tagging single nucleotide polymorphisms (SNPs) in *TNF* and *LTA,* including functional variants, in 596 nuclear families consisting of asthmatics 4–17 years of age and their parents in Mexico City. Atopy was determined by skin prick tests.

**Results:**

The A allele of the *TNF*-308 SNP was associated with increased risk of asthma [relative risk (RR) = 1.54; 95% confidence interval (CI), 1.04–2.28], especially among children of non-smoking parents (RR = 2.06; 95% CI, 1.19–3.55; *p* for interaction = 0.09). Similarly, the A allele of the *TNF*-238 SNP was associated with increased asthma risk among children of nonsmoking parents (RR = 2.21; 95% CI, 1.14–4.30; *p* for interaction = 0.01). *LTA* SNPs were not associated with asthma. Haplotype analyses reflected the single SNP findings in magnitude and direction. *TNF* and *LTA* SNPs were not associated with the degree of atopy.

**Conclusions:**

Our results suggest that genetic variation in *TNF* may contribute to childhood asthma and that associations may be modified by parental smoking.

Asthma is a complex disease characterized by airway inflammation, bronchial hyper-responsiveness, and airflow obstruction. Tumor necrosis factor-α (TNF), the defining member of the TNF family of cytokines, has been directly implicated in asthmatic airway inflammation and bronchial hyperresponsiveness (Babu et al. 2004; Thomas 2001). Lymphotoxin-α (LTA), also called TNF-β, shares receptors with TNF. The *TNF* [GenBank accession no. X02910 (http://www.ncbi.nlm.nih.gov/entrez/viewer.fcgi?val=X02910)] and *LTA* [GenBank accession no. X01393 (http://www.ncbi.nlm.nih.gov/entrez/viewer.fcgi?val=X01393)] genes are located consecutively in the class III region of the human major histocompatibility complex (MHC) on chromosome 6p21, which has shown evidence of linkage to asthma, atopy, and related phenotypes in multiple genome-wide studies (Collaborative Study on the Genetics of Asthma 1997; Daniels et al. 1996).

Single nucleotide polymorphisms (SNPs) of both *TNF* and *LTA* influence gene expression. (Knight et al. 2004; Kroeger et al. 1997; Messer et al. 1991; Ozaki et al. 2002; Wilson et al. 1997). In particular, a SNP in the *TNF* promoter region (*TNF*-308) and a SNP within the first intron of *LTA* (*LTA Nco*I) affect the rate of gene transcription and protein production (Kroeger et al. 1997; Messer et al. 1991; Ozaki et al. 2002; Wilson et al. 1997). Several studies have indicated an association of the *TNF*-308 SNP with asthma and atopy susceptibility (Albuquerque et al. 1998; Bilolikar et al. 2005; Chagani et al. 1999; Di Somma et al. 2003; Gao et al. 2006; Gupta et al. 2005; Li Kam Wa et al. 1999; Li YF et al. 2006; Moffatt and Cookson 1997; Sandford et al. 2004; Sharma et al. 2006; Shin et al. 2004; Wang et al. 2004; Winchester et al. 2000; Witte et al. 2002), although other studies do not (Beghe et al. 2004; Buckova et al. 2002; El Bahlawan et al. 2003; Lin et al. 2002; Louis et al. 2000; Moffatt et al. 1999; Randolph et al. 2005; Trabetti et al. 1999; Zhu et al. 2000). The *LTA Nco*I SNP was reported to be associated with asthma (Albuquerque et al. 1998; Bilolikar et al. 2005; Moffatt and Cookson 1997; Sharma et al. 2006); however, most studies showed no association (Buckova et al. 2002; Cardaba et al. 2001; El Bahlawan et al. 2003; Immervoll et al. 2001; Izakovicova Holla et al. 2001; Li Kam Wa et al. 1999; Lin et al. 2002; Migita et al. 2005; Moffatt et al. 1999; Noguchi et al. 2002; Randolph et al. 2005; Sandford et al. 2004; Shin et al. 2004; Trabetti et al. 1999; Van Hage-Hamsten et al. 2002; Wang et al. 2004; Witte et al. 2002). The *TNF*-857 and *LTA*-753 SNPs in the promoter regions have also been associated with asthma and atopy (Migita et al. 2005; Noguchi et al. 2002). The effects of *TNF* and *LTA* SNPs on asthma and atopy remain unresolved due to the conflicting results across studies.

The etiology of asthma and atopy involves interactions between genetic susceptibility and exposure to environmental triggers, such as secondhand smoke, ozone, particulate matter, allergens, and endotoxin (Tatum and Shapiro 2005). *TNF* has been identified as a candidate gene for ozone-induced airway inflammation and hyperresponsiveness (Kleeberger et al. 1997), and genetic variation in *TNF* and *LTA* has been associated with respiratory effects of ozone in humans (Yang et al. 2005). Parental smoking has been consistently related to childhood asthma [U.S. Department of Health and Human Services (DHHS) 2006] and TNF may influence the lung inflammatory response to tobacco smoke (Churg et al. 2003; Park et al. 2003). Few studies have examined whether exposure to parental smoking modifies the relationship between *TNF* and *LTA* polymorphisms and asthma risk. We used the case–parent triad design to investigate the association of *TNF* and *LTA* polymorphisms and haplotypes with childhood asthma and atopy in asthmatic children from Mexico City, an area with the highest ozone exposure in North America. We also examined possible effect modification by exposure to parental smoking.

## Methods

### Study design and subject enrollment

We used the case–parent triad design (Weinberg et al. 1998; Wilcox et al. 1998). The study population included 536 case–parent triads and 60 case–parent pairs with adequate DNA samples for genotyping of at least one SNP for the *TNF* and *LTA* genes. The cases were children 4–17 years of age with asthma diagnosed by a pediatric allergist at the allergy referral clinic of a large public pediatric hospital in central Mexico City (Hospital Infantil de Mexico Federico Gomez). Children and parents provided blood samples as sources of DNA. A parent, nearly always the mother, completed a questionnaire on the child’s symptoms and risk factors for asthma including current parental smoking, parental smoking during the first 2 years of the child’s life, maternal smoking during pregnancy, and residential history.

We obtained measurements of ambient ozone from the Mexican government’s air monitoring stations (http://www.ine.gob.mx/dgicurg/calaire/tend/concentra.php). Ozone levels were measured via ultraviolet photometry (analyzer model 400, Advanced Pollution Instrumentation, San Diego, CA, USA). The residence of each child who participated in this study was located using a map, and the closest monitoring station was assigned to that residence (Romieu et al. 2002). The ozone exposure data were collected for the year before the time of entry into the study. The parameter we used was the annual average of the daily maximum 8-hr averages. We dichotomized this variable at the median of 67 ppb for stratified analyses.

The protocol was reviewed and approved by the institutional review boards of the Mexican National Institute of Public Health, the Hospital Infantil de Mexico Federico Gomez, and the U.S. National Institute of Environmental Health Sciences (NIEHS). Parents provided the written informed consent for the child’s participation. Children also gave their informed assent.

### Clinical evaluation

The diagnosis of asthma was based on clinical symptoms and response to treatment by a pediatric allergist [British Thoracic Society/Scottish Intercollegiate Guidelines Network (BTS/SIGN) 2003]. The severity of asthma was rated by a pediatric allergist for 571 cases according to symptoms in the Global Initiative on Asthma schema as mild (intermittent or persistent), moderate, or severe [National Heart, Lung, and Blood Institute (NHLBI) 1998]. At a different point of time, for research purposes, pulmonary function was measured using the EasyOne spirometer (ndd Medical Technologies, Andover, MA, USA) for 446 cases according to American Thoracic Society (ATS) specifications (ATS 1995). The best test of three technically acceptable tests was selected. Spirometric prediction equations from a Mexico City childhood population were used to calculate the percent predicted forced expiratory volume in 1 sec (FEV_1_) (Perez-Padilla et al. 2003). Children were asked to hold asthma medications on the morning of the test.

Atopy was determined using skin prick tests. The following battery of 24 aeroallergens common in Mexico City was used: *Aspergillus fumigatus*, *Alternaria*, *Mucor*, *Blattella germanica*, *Periplaneta americana*, *Penicillium*, cat, dog, horse, *Dermatophagoides (pteronyssinus and farina)*, *Ambrosia*, *Artemisa ludoviciana*, *Cynodon dactylon*, *Chenopodium album*, *Quercus*, *Fraxinus*, *Helianthus*, *Ligustrum vulgare*, *Lolium perenne*, *Plantago lanceolata*, *Rumex crispus*, *Schinus molle*, *Salsola*, and *Phleum pratense*. Histamine was used as a positive control and glycerin as a negative control. Children were considered atopic if the diameter of the skin reaction to at least one allergen exceeded 4 mm. The test was considered valid if the reaction to histamine was ≥ 6 mm according to the grading of skin prick test recommended by Aas and Belin (1973). Skin test data on all 24 aeroallergens were available on 545 cases.

### SNP selection

We had various data sources available for selection of tagging SNPs. These included resequencing data in individuals of African and European descent by Seattle SNPs (http://pga.mbt.washington.edu) and genotyping data on 10 Mexicans and 38 Mexican-Americans with four grandparents born in Mexico for cosmopolitan haplotype tagging SNPs identified by the NIEHS Environmental Genome Project based on resequencing a representative sample of the U.S. population (http://egp.gs.washington.edu). We identified the common haplotypes using PHASE (Stephens et al. 2001) and then used ldSelect (Carlson et al. 2004) to identify tagging SNPs. We also analyzed the genotyping data from seven Mexicans in SNP500Cancer (Packer et al. 2004) (http://snp500cancer.nci.nih.gov). *TNF* and *LTA* are small genes located consecutively in a 6 kb region on chromosome 6, and linkage disequilibrium in this region is high. We selected six tagging SNPs—*TNF*-1031 (rs1799964), *TNF*-857 (rs1799724), *TNF*-308 (rs1800629), *TNF*-238 (rs361525), *LTA*-379 (rs2239704), and *LTA NcoI* (rs909253)—to cover the whole region, including all the common SNPs in the regulatory and coding regions of the genes with known functional importance (Fong and Mark 1995; Knight et al. 2004; Kroeger et al. 1997; Messer et al. 1991; Ozaki et al. 2002; Wilson et al. 1997) or that had been associated with asthma in the literature. As expected, given the increasing documentation of the portability of tagging SNPs across populations (Gonzalez-Neira et al. 2006), especially in the less diverse non-African groups, the tagging SNPs selected provide excellent coverage of common haplotypes in other populations. For example, the six tagging SNPs we selected based on the Mexican data would cover all six common haplotypes (> 5%) in European and five of six common haplotypes in African populations.

### Genotyping

We extracted DNA from peripheral blood lymphocyte using Gentra Puregene kits (Gentra System, Minneapolis, MN, USA). We obtained genotypes for the *TNF*-1031 and *TNF*-857 SNPs using TaqMan SNP Genotyping Assay (Li H et al. 2006). Primers and probes were purchased from Assay-on-Demand (Applied Biosystems, Foster City, CA, USA). All PCR amplifications were performed using 5′ exonuclease assay on GeneAmp PCR Systems 9700 (Applied Biosystems). The fluorescence of PCR products was detected using ABI Prism 7900HT sequence detection system. The *TNF*-308, *TNF*-238, and *LTA Nco*I SNPs were genotyped using a multiplex PCR and immobilized probe linear array system (Barcellos et al. 2004), provided by Roche Molecular Systems (Alameda, CA, USA). The *LTA*-379 SNP was genotyped using MGB Eclipse Genotyping Assay (Belousov et al. 2004). Primers and Probes were purchased from MGB Eclipse by Design (Epoch Biosciences, Bothell, WA, USA). All genotyping assays were done by a researcher who was blinded to parent or child status of samples. Sixteen quality control samples were plated per 384-well plate along with 24 control samples with known genotype. An additional six blind replicate samples were included in the analyses. The quality controls and the blind replicates were 100% concordant for all genotyping methods.

Nonparentage was ascertained with a set of short-tandem repeats (AmpFLSTR Profiler Plus; Applied Biosystems) analyzed using Pedcheck software (University of Pittsburgh, Pittsburgh, PA, USA) (O’Connell and Weeks 1998). A total of 596 families had genotyping data for at least one SNP, and 566 families had genotyping data for all six SNPs.

### Statistical analysis

We used a log-linear likelihood approach to analyze associations between asthma and individual SNPs (Weinberg et al. 1998). The log-linear likelihood-ratio test is a powerful and more flexible alternative to the transmission disequilibrium test (TDT) and tests the same null hypothesis of no within-family relationship between the variant and the disease (Lake and Laird 2004). Similar to TDT-based methods for the analysis of case–parent data, such as the family-based association test (FBAT) (Horvath et al. 2001), the log-linear model achieves robustness against genetic population structure through stratification on the possible parental mating types (Lake and Laird 2004). The log-linear method has the advantage of providing estimates of the magnitude of associations rather than simply tests of significance (Weinberg et al. 1998). We calculated relative risks for individual SNPs without restricting to a specific genetic model. The log-linear models of case–parent data are inherently immune to confounding by demographic or lifestyle factors such as parental smoking. However, we examined effect modification by sex, asthma severity, parental smoking, and level of ozone exposure. We calculated tests of interactions for the joint effects of genotype and current parental smoking and parental smoking before the child turned two using the method of Umbach and Weinberg (2000). All analyses were performed using SAS version 9.1 (SAS Institute Inc., Cary, NC, USA) and STATA version 8.0 (StataCorp., College Station, TX, USA).

To evaluate whether *TNF* and *LTA* polymorphisms influenced the degree of atopy, as assessed by the number of positive skin tests out of 24 performed, we used the polytomous logistic method of Kistner and Weinberg to estimate the linkage and association between *TNF* and *LTA* polymorphisms and atopy (Kistner and Weinberg 2004). *p*-Values were calculated from likelihood ratio tests. We also used this method to analyze the relationship between *TNF* and *LTA* SNPs and lung function, as assessed by percent predicted FEV_1_.

We used HAPLIN version 2.0 (http://www.uib.no/smis/gjessing/genetics/software/haplin) to analyze associations between asthma and *TNF* and *LTA* haplotypes. HAPLIN is an extension of the log-linear model from a single locus to loci with multiple haplotypes with unknown phase (Gjessing and Lie 2005). The haplotypes of individuals with unknown phase are constructed from the family information whenever possible, and the remaining haplotypes are estimated by using the expectation-maximization algorithm (Gjessing and Lie 2005). HAPLIN estimates single- and double-dose effects of haplotypes rather than simply tests of significance using maximum likelihood (Gjessing and Lie 2005). We set a threshold of 1% for haplotype frequency, leaving 582 families in the HAPLIN analyses.

We present the *p*-values computed using the above methods. To address the potential issue of multiple comparisons, we calculated the false discovery rate for each *p*-value < 0.05 using the method of Storey (Storey and Tibshirani 2003). The false discovery rate is the expected proportion of false positives incurred when a particular test is called significant. However, these corrections will be overly conservative when applied equally to all SNPs. Our prior prediction of positive findings would be greatest for SNPs with known functional importance, such as *TNF*-308, compared with SNPs chosen only as haplotype tagging SNPs where functional significance is not well characterized. In addition, the false discovery rate does not take into account the correlation between SNPs in a gene.

## Results

Clinical characteristics of the asthmatic children with genotyping data are presented in Table 1. The mean (± SD) age of cases was 9.0 ± 2.4 years (range 4–17 years). Most had mild (71.5%) as opposed to moderate or severe asthma (28.5%). Nearly all cases (98.3%) had used medication for asthma in the preceding 12 months. Wheezing in the preceding 12 months was reported by 89.8% and chronic dry cough was reported by 65.4%. For 73.9% of cases, asthma symptoms had interfered with daily activities or school attendance in the preceding 12 months. Among cases with spirometry data, the mean FEV_1_ percent predicted was 96.7 ± 20.6. Atopy was present in 91.9% of cases. The highest rates of skin test positivity were seen for dust mite (70.3%) and cockroach (43.1%). Although only 5.8% of mothers reported smoking during pregnancy, 32.6% of cases lived with a smoking parent in early childhood (before 2 years of age) and 50.4% were currently exposed to parental smoking.

The minor allele frequency and genotype frequency distributions of the six tagging SNPs are shown in Table 2. The *TNF*-308 and *TNF*-238 polymorphisms were relatively rare in our Mexican population (minor allele frequency = 5% for *TNF*-308 and 4% for *TNF*-238). The frequency distributions for all mating types for *TNF* and *LTA* polymorphisms are presented in Supplemental Table 1 for all families, Supplemental Table 2 for families with smoking parents, and Supplemental Table 3 for families with non-smoking parents (Supplemental Material online at http://www.ehponline.org/docs/2007/9740/suppl.pdf). Hardy-Weinberg equilibrium (*p* > 0.1) was confirmed for all six tagging SNPs in the parents. Pairwise linkage disequilibrium coefficients, *D*, and *r* ^2^, between *LTA* and *TNF* SNPs, calculated using FBAT (Horvath et al. 2001) are shown in Supplemental Table 4 in the online Supplemental Material. There was moderate linkage disequilibrium between the *LTA*-379 and *LTA Nco*I polymorphisms (*r* ^2^ = 0.50), and weak linkage disequilibrium between the *LTA*-379 and *TNF*-857 polymorphisms (*r* ^2^ = 0.39), the *TNF*-1031 and *TNF*-238 polymorphisms (*r* ^2^ = 0.25), the *LTA Nco*I and *TNF*-857 polymorphisms (*r* ^2^ = 0.20), and the *LTA*-379 and *TNF*-1031 polymorphisms (*r*^2^ = 0.16). The *r*^2^ values for all other SNP pairs were < 0.1.

Neither of the two SNPs in *LTA* was associated with asthma (Table 3). For *TNF*, carrying at least one copy of the *TNF*-308A allele was associated with increased asthma risk [relative risk (RR) = 1.54; 95% confidence interval (CI), 1.04–2.28; *p* = 0.031; false discovery rate = 0.12] relative to homozygotes for the major G allele (Table 3). Only three cases were homozygous for the minor allele for *TNF*-238 and none for *TNF*-308, so we were not able to evaluate the relative risks for homozygotes. The *TNF*-238, *TNF*-857, and *TNF*-1031 polymorphisms gave weaker, and more unstable, evidence of association with asthma (Table 3). Results for the six tagging SNPs did not differ appreciably by sex, asthma severity (mild versus moderate to severe), or ozone level (data not shown). We did not observe a clear pattern of associations between the *TNF* and *LTA* SNPs and percent predicted FEV_1_ (data not shown).

Because of the consistent association between parental smoking and childhood asthma (DHHS 2006) and the involvement of TNF in cigarette smoke–induced inflammation responses (Churg et al. 2003; Park et al. 2003), we examined the association of each *LTA* and *TNF* SNP with asthma stratified by exposure to a smoking parent in the home. Among cases without smoking parents in the home, the *TNF*-308A allele and *TNF*-238A alleles showed increased risk of asthma (RR = 2.06; 95% CI, 1.19–3.55; *p* = 0.0097; false discovery rate = 0.04 for *TNF*-308A; RR = 2.21; 95% CI, 1.14–4.30; *p* = 0.019; false discovery rate = 0.04 for *TNF*-238A) (Table 3). The *p*-values for interaction with living with smoking parents were 0.09 for *TNF*-308 and 0.01 for *TNF*-238. For the other two *TNF* SNPs, although the results were not statistically significant, the increased risk of asthma for carrying two copies of the minor allele also appeared to be limited to cases with nonsmoking parents (RR = 2.36; 95% CI, 0.82–6.78 for *TNF*-1031C; RR = 1.32; 95% CI, 0.76–2.29 for *TNF*-857T). Among cases with smoking parents in the home, none of the six *LTA* and *TNF* polymorphisms were associated with asthma (Table 3).

Haplotype analyses results reflected the single SNP findings in magnitude and direction (Table 4). Among all cases, individuals carrying one copy of the ht5 (CGTCAG) haplotype, containing the *TNF*-308A allele, exhibited an increased risk of asthma of borderline statistical significance (RR = 1.45; 95% CI, 0.97–2.19). Among cases with non-smoking parents, carrying one copy of the ht6 (CACCGA) haplotype containing the *TNF*-238A allele or one copy of the ht5 haplotype exhibited an increased risk of asthma (RR = 2.15; 95% CI, 1.21–3.82; *p* = 0.0082 for ht5; RR = 2.40; 95% CI, 1.18–4.81; *p* = 0.014 for ht6). The false discovery rate was 0.03 for the ht5 and ht6 findings. Among cases with smoking parents, *LTA* and *TNF* haplotypes were not associated with asthma (Table 4).

We also examined the association of *LTA* and *TNF* individual SNPs and haplotypes with asthma stratified by exposure to a smoking parent before child turned two because exposure in early childhood has also been consistently associated with childhood asthma risk (DHHS 2006). The analysis results were consistent with findings for stratifying by current exposure to a smoking parent in the home (data not shown).

We examined the association between individual *LTA* and *TNF* SNPs and the degree of atopy to aeroallergens, assessed by the number of positive skin tests out of a battery of 24 tests. No significant associations were detected with the number of positive skin tests for the six *LTA* and *TNF* SNPs (data not shown).

## Discussion

In this case–parent triad study in a Mexico City population with high lifetime exposure to ozone, we found that the A allele of the functionally relevant *TNF*-308 polymorphism was significantly associated with an increased risk of childhood asthma, especially among children with nonsmoking parents. The *TNF*-238A allele and the haplotypes containing the *TNF*-308A allele or the *TNF*-238A allele were associated with an increased childhood asthma risk predominantly in children with nonsmoking parents.

TNF is a potent proinflammation cytokine and has been consistently implicated in asthmatic inflammation and bronchial hyperresponsiveness in a variety of subcellular, *in vitro*, *ex vivo*, *in vivo*, and genetic studies (Thomas 2001). For example, TNF expression is markedly increased in asthmatic airways compared with normal airways (Bradding et al. 1994). TNF appears to have an important amplifying effect on asthmatic inflammation (Babu et al. 2004) and has also been shown to induce airway hyperresponsiveness in rats and humans (Kips et al. 1992; Thomas et al. 1995). Therefore, genetic polymorphisms that affect gene expression or TNF activity in the airways might be expected to influence asthma risk.

The *TNF*-308 polymorphism has been frequently studied in asthma and atopy association studies because it has direct functional effects on *TNF* gene regulation. The *TNF*-308A allele is a much stronger transcriptional activator than the more common G allele (Wilson et al. 1997) and is associated with higher TNF production (Louis et al. 1998). The *TNF*-308A allele leads to high binding affinity of nuclear factors to the *TNF* promoter and gives a high level of gene transcription (Kroeger et al. 1997). Thus, observations from functional studies suggest that the *TNF*-308A allele is of biological significance. Despite the known effects of tobacco smoke on TNF expression and the well-documented association between parental smoking and childhood asthma, few studies have evaluated whether exposure to a smoking parent modifies effects of genetic variation in *TNF* on childhood asthma. In our study, the association was greater among children without smoking parents in the home.

We also found an association between the *TNF*-238A allele and asthma risk among children with nonsmoking parents in the home. The *TNF*-238 polymorphism was not in linkage disequilibrium with the *TNF*-308 polymorphism (*r*^2^ < 0.01) in our Mexican population, although they are only 70 bp apart from each other within the class III region of the MHC on chromosome 6p. There is no strong evidence showing that the *TNF*-238 polymorphism has a direct effect on gene expression, although studies suggest that this region contains a strong repressor site (−280 to −172) (Fong and Mark 1995). However, the *TNF*-238 polymorphism may be in linkage disequilibrium with a functional polymorphism that impacts TNF production, either within the *TNF* gene or another gene within the MHC.

Because the etiology of asthma involves numerous environmental triggers, heterogeneous exposure to environmental stimuli among different populations may cause conflicting results across studies. The influence of genotypes on phenotypes may be different and even opposite at different levels of exposure (Martinez 2005). Therefore, incorporating environmental exposures, such as parental smoking, into association studies is important. Parental smoking, a reasonably valid approach to estimate long-term secondhand smoke exposure in infants and children, is one of the most consistent risk factors for childhood asthma (DHHS 2006).

It is not unexpected to find differing associations between genetic polymorphisms and asthma susceptibility based on parental smoking exposure. We previously reported that the protective effect for the NQO1 Ser allele in GSTM1-null children was limited to those with nonsmoking parents (David et al. 2003). A recent genome-wide linkage study found different regions of linkage to childhood asthma by parental smoking exposure (Colilla et al. 2003). Of note, the chromosome 6p region, containing the *TNF* gene, was more strongly linked to asthma among subjects who did not live with a smoker during infancy compared with those who lived with smokers (Colilla et al. 2003).

A recent study showed that the *TNF*-308 polymorphism modified the effect of home exposure to smokers on respiratory illness-related school absence among children mostly without asthma (Wenten et al. 2005). Another study in the same population showed that the *TNF*-308A allele was associated with an increased risk of wheezing, especially among children living in low ozone communities (Li YF et al. 2006). In the present study, we found that *TNF* polymorphisms and haplotypes were associated with childhood asthma susceptibility predominantly among children who did not live with smoking parents.

Because *TNF* is a candidate gene for ozone-induced airway inflammation and hyperresponsiveness (Kleeberger et al. 1997), we stratified on the ozone level. It is not surprising that we did not observe effect modification by ozone because our population in central Mexico City was exposed to high lifetime levels of ozone compared with U.S. populations. The median level of the annual average of the daily maximum 8-hr averages for our population was 67 ppb, with an interquartile range of only 12 ppb.

A possible explanation for our finding of an association of *TNF* polymorphisms with asthma predominantly for children without smoking parents is that the combination of exposure to secondhand smoke and ozone, which both increase TNF production, overwhelms the smaller impact of *TNF* polymorphisms on TNF expression. Indeed, expression levels of TNF are significantly increased in mice and humans exposed to tobacco smoke (Churg et al. 2003; Park et al. 2003). TNF has been identified as a potential candidate gene for ozone susceptibility (Kleeberger et al. 1997; Yang et al. 2005) and ozone exposure can stimulate TNF secretion from lung cells (Arsalane et al. 1995). Tobacco smoke and ozone are both strong oxidants (Tatum and Shapiro 2005) and potent controllers of TNF production (Arsalane et al. 1995; Churg et al. 2003; Park et al. 2003). In combination, these environmental triggers may have synergistic effects and overcome the smaller effects of *TNF* polymorphisms. Conversely, in the absence of secondhand smoke exposure, the influence of *TNF* functional polymorphisms on TNF expression may be greater, which could explain our finding of stronger associations at lower levels of exposure. Of note, for CD14, a pattern recognition receptor in the endotoxin-induced immune response, associations between the *CD14*-159 polymorphism and asthma and atopy differ at high versus low levels of endotoxin exposure (Martinez 2005).

In summary, we found that *TNF* polymorphisms and haplotypes were associated with childhood asthma susceptibility, especially among children without smoking parents. These results suggest that the effects of genetic variation in *TNF* may be more apparent at lower levels of exposure to substances such as secondhand smoke, which strongly influence TNF expression.

## Figures and Tables

**Table 1:** Demographic and clinical characteristics of the 596 asthmatic children.

Clinical characteristics	Value
Age [years (mean ± SD)]	9.0 ± 2.4
Sex (male)	61.1
Asthma severity (*n* = 571)	
Mild	71.5
Moderate to severe	28.5
Asthma medication in the preceding 12 months (*n* = 590)	98.3
FEV_1_ [percent predicted (mean ± SD)] (*n* = 446)	96.7 ± 20.6
Skin test positivity (of 24 aeroallergens) (*n* = 545)	
≥ 1 allergen	91.9
≥ 5 allergens	52.8
Parental smoking (*n* = 591)	
Mother smoked during pregnancy	5.8
In early childhood (< 2 years of age)	32.6
Current smoking parent	50.4

Values are expressed as percent except where noted.

**Table 2:** Genotype distributions for *LTA* and *TNF* polymorphisms.

Locus	Genotype	All cases	No. of cases with smoking parents	No. of cases with nonsmoking parents	Minor allele frequency^a^
*LTA*-379	CC	159	85	74	0.48
	CA	299	147	148	
	AA	138	66	71	
*LTA Nco*I	AA	251	121	128	0.34
	AG	268	141	124	
	GG	65	32	33	
*TNF*-1031	TT	420	208	208	0.15
	TC	146	74	71	
	CC	18	9	9	
*TNF*-857	CC	316	165	150	0.27
	CT	221	106	111	
	TT	50	21	29	
*TNF*-308	GG	513	268	240	0.05
	GA	65	25	40	
	AA	0	0	0	
*TNF*-238	GG	525	269	252	0.04
	GA	50	22	28	
	AA	3	2	1	

See the Supplemental Material (http://www.ehponline.org/docs/2007/9740/suppl.pdf) for the frequency distributions for all mating types for *TNF* and *LTA* polymorphisms for all families and by parental smoking status.

a Minor allele frequency was calculated using parent genotyping data.

**Table 3:** *LTA* and *TNF* polymorphisms in relation to childhood asthma risk among all cases and stratified by parental smoking status [RR (95% CI)].

Locus	Genotype	All cases	Cases with smoking parents	Cases with nonsmoking parents
*LTA*-379	CC	1.0	1.0	1.0
	CA	1.04 (0.82–1.32)	0.95 (0.68–1.31)	1.11 (0.78–1.57)
	AA	0.98 (0.71–1.34)	0.93 (0.59–1.45)	1.02 (0.65–1.62)
*LTA Nco*I	AA	1.0	1.0	1.0
	AG	1.00 (0.80–1.24)	1.04 (0.76–1.43)	0.94 (0.68–1.30)
	GG	0.89 (0.61–1.29)	1.00 (0.60–1.69)	0.79 (0.46–1.35)
*TNF*-1031	TT	1.0	1.0	1.0
	TC	1.01 (0.78–1.30)	0.89 (0.61–1.30)	1.14 (0.80–1.64)
	CC	1.22 (0.64–2.33)	0.87 (0.38–2.00)	2.36 (0.82–6.78)
*TNF*-857	CC	1.0	1.0	1.0
	CT	0.96 (0.77–1.19)	0.88 (0.64–1.20)	0.99 (0.73–1.35)
	TT	1.13 (0.75–1.70)	0.93 (0.51–1.71)	1.32 (0.76–2.29)
*TNF*-308	GG	1.0	1.0	1.0
	GA	1.54 (1.04–2.28)*	1.09 (0.61–1.94)	2.06 (1.19–3.55)**
	AA^a^	—	—	—
*TNF*-238	GG	1.0	1.0	1.0
	GA	1.22 (0.81–1.85)	0.81 (0.46–1.42)	2.21 (1.14–4.30)#
	AA^a^	—	—	—

a Only three cases were homozygous for the minor A allele for *TNF*-238 and none for *TNF*-308, so we did not calculate those relative risks.

* *p* = 0.031; false discovery rate = 0.12.

** *p* = 0.0097; false discovery rate = 0.04; *p*-value for interaction = 0.09.

# *p* = 0.019; false discovery rate = 0.04; *p*-value for interaction = 0.01.

**Table 4:** *TNF* and *LTA* haplotypes in relation to childhood asthma risk among all cases and stratified by parental smoking status [RR (95% CI)].

Haplotype^a^	Frequency	Single copy	Double copy
All cases			
ht1 (CGTCGG)	0.305	0.94 (0.75–1.18)	0.80 (0.53–1.21)
ht2 (AATTGG)	0.271	0.92 (0.72–1.15)	1.11 (0.73–1.67)
ht3 (AATCGG)	0.208	1.00 (0.79–1.26)	1.00 (0.60–1.66)
ht4 (CACCGG)	0.112	0.87 (0.65–1.16)	1.04 (0.47–2.33)
ht5 (CGTCAG)	0.040	1.45 (0.97–2.19)	—b
ht6 (CACCGA)	0.035	1.31 (0.85–2.03)	—
ht7 (CATCGG)	0.027	1.03 (0.62–1.73)	—
Cases with smoking parents			
ht1 (CGTCGG)	0.298	1.18 (0.86–1.63)	1.02 (0.57–1.81)
ht2 (AATTGG)	0.271	0.86 (0.62–1.21)	0.89 (0.49–1.66)
ht3 (AATCGG)	0.198	1.09 (0.78–1.54)	1.30 (0.65–2.63)
ht4 (CACCGG)	0.117	0.88 (0.58–1.31)	0.98 (0.32–3.03)
ht5 (CGTCAG)	0.042	0.90 (0.49–1.67)	—
ht6 (CACCGA)	0.047	0.84 (0.46–1.52)	—
ht7 (CATCGG)	0.022	1.50 (0.72–3.15)	—
Cases with nonsmoking parents			
ht1 (CGTCGG)	0.310	0.74 (0.54–1.02)	0.64 (0.36–1.16)
ht2 (AATTGG)	0.274	0.92 (0.66–1.27)	1.29 (0.72–2.22)
ht3 (AATCGG)	0.212	0.94 (0.66–1.31)	0.81 (0.38–1.73)
ht4 (CACCGG)	0.107	0.86 (0.57–1.31)	1.16 (0.37–3.53)
ht5 (CGTCAG)	0.038	2.15 (1.21–3.82)*	—
ht6 (CACCGA)	0.022	2.40 (1.18–4.81)**	—
ht7 (CATCGG)	0.032	0.71 (0.34–1.49)	—

a The haplotypes formed by *LTA*-379C > A, *LTA Nco*IA > G, *TNF*-1031T > C, *TNF*-857C > T, *TNF*-308G > A, and *TNF*-238G > A in order.

b Only three cases were homozygous for the ht6 haplotype and none for the ht5 and ht7 haplotypes, so we did not calculate those relative risks.

* *p* = 0.0082; false discovery rate = 0.03.

** *p* = 0.014; false discovery rate = 0.03.
